# Development and Validation of a Nomogram for Predicting the Risk of Bell's Stage II/III Necrotizing Enterocolitis in Neonates Compared to Bell's Stage I

**DOI:** 10.3389/fped.2022.863719

**Published:** 2022-06-14

**Authors:** Shuting Song, Jian Zhang, Yuwei Zhao, Liying Dai

**Affiliations:** Department of Neonatology, Anhui Provincial Children's Hospital, Anhui Medical University, Hefei, China

**Keywords:** necrotizing enterocolitis, neonate, nomogram, risk factors, prediction model

## Abstract

**Background:**

Patients with Bell's Stage II/III necrotizing enterocolitis (NEC) may have more severe presentations, higher rates of death, and more long-term complications than those with Bell's Stage I NEC, so the purpose of this article was to construct a nomogram model to distinguish Bell's stage II/III NEC early from Bell's Stage I NEC, which is critical in the clinical management of NEC.

**Patients and Methods:**

A total of 730 NEC newborns diagnosed from January 2015 to January 2021 were retrospectively studied. They were randomly divided into training and validation groups at the ratio of 7:3. A nomogram model for predicting NEC was developed based on all the independent risk factors by multivariate regression analysis. The model's performance was mainly evaluated through three aspects: the area under the curve (AUC) to verify discrimination, the Hosmer–Lemeshow test and calibration curve to validate the consistency, and decision curve analysis (DCA) to determine the clinical effectiveness.

**Results:**

Predictors included in the prediction model were gestational age (GA), birth weight (BW), asphyxia, septicemia, hypoglycemia, and patent ductus arteriosus (PDA). This nomogram model containing the above-mentioned six risk factors had good discrimination ability in both groups, and the AUCs were 0.853 (95% CI, 0.82–0.89) and 0.846 (95% CI, 0.79–0.90), respectively. The calibration curve and DCA confirmed that the nomogram had good consistency and clinical usefulness.

**Conclusions:**

This individual prediction nomogram based on GA, BW, asphyxia, septicemia, hypoglycemia, and PDA served as a useful tool to risk-stratify patients with NEC, and can help neonatologists early distinguish Bell's stage II/III NEC early from Bell's Stage I NEC.

## Introduction

Necrotizing enterocolitis (NEC) is a serious gastrointestinal disease in the neonatal intensive care units (NICUs) and is a major contributor to mortality in premature infants. Despite continuous improvements in medical technology, the morbidity and mortality rates are still as high as 7–13% and 10–40% (1, 2), and the survivors are likely to have long-term complications, such as short bowel syndrome, intestinal strictures or adhesions, cholestasis, and neurodevelopmental delays (3, 4). Moreover, stage II/III NEC tend to be more likely to have complications and have higher mortality and disability rates. Thus, it is crucial to establish a risk stratification assessment tool for NEC.

The etiology of NEC is thought to be multifactorial. However, the role of a single factor with NEC needs to be further elucidated. Studies have shown that NEC mainly occurs in 90–95% of infants <36 weeks of gestational age (GA) (5). A multi-center study of epidemiological data showed that the incidence of NEC is negatively correlated with birth weight (BW). The incidence of NEC is approximately 2.50% in low-birth-weight infants (<2,500 g), and the incidence is approximately 4.53% in very-low-birth-weight infants (<1,500 g) (6). In addition to the aforementioned GA and BW being recognized risk factors, some of the previous studies have also identified other independent risk factors, such as septicemia, patent ductus arteriosus (PDA), erythrocyte infusions, and formula feeding (7, 8) for the occurrence of NEC. However, traditional statistical methods can only determine the risk factors for the occurrence of the outcomes. These methods cannot predict the probability of the occurrence of NEC. This article analyzed the clinical data of NEC patients to find out the risk factors for NEC. Then, a prediction model was established based on these findings. Finally, an internal validation was performed based on this model by using the data in the verification group so that the model can be better used to predict the probability of NEC in other individuals in the future. The previous research on the establishment of prediction models for NEC have mainly focused on the establishment of a surgical risk prediction model and a death risk factor scoring system model for NEC (9, 10), but a risk stratification assessment model for NEC has not yet been established.

The nomogram is one of the most representative models of all the prediction models, and the nomogram is more accurate and has better performance characteristics than artificial neural networks (ANNs), classification and regression tree models (CARTs), look-up tables, and risk-group stratification models (11). For example, the nomogram Zhang et al. (12) developed can nicely and relatively accurately predict the probability of Bronchopulmonary Dysplasia (BPD) in infants with a BW <1,500 g, reducing the chances of exposure to risk factors could decrease the incidence of BPD. Therefore, the nomogram is really a simple and useful model to predict the risk of clinical disease. As a result, developing a predictive nomogram model of NEC will be promising for distinguishing Bell's stage II/III NEC early from Bell's Stage I NEC, and taking early specific interventions to reduce the probability of adverse outcomes.

## Patients and Methods

**Figure 1:**
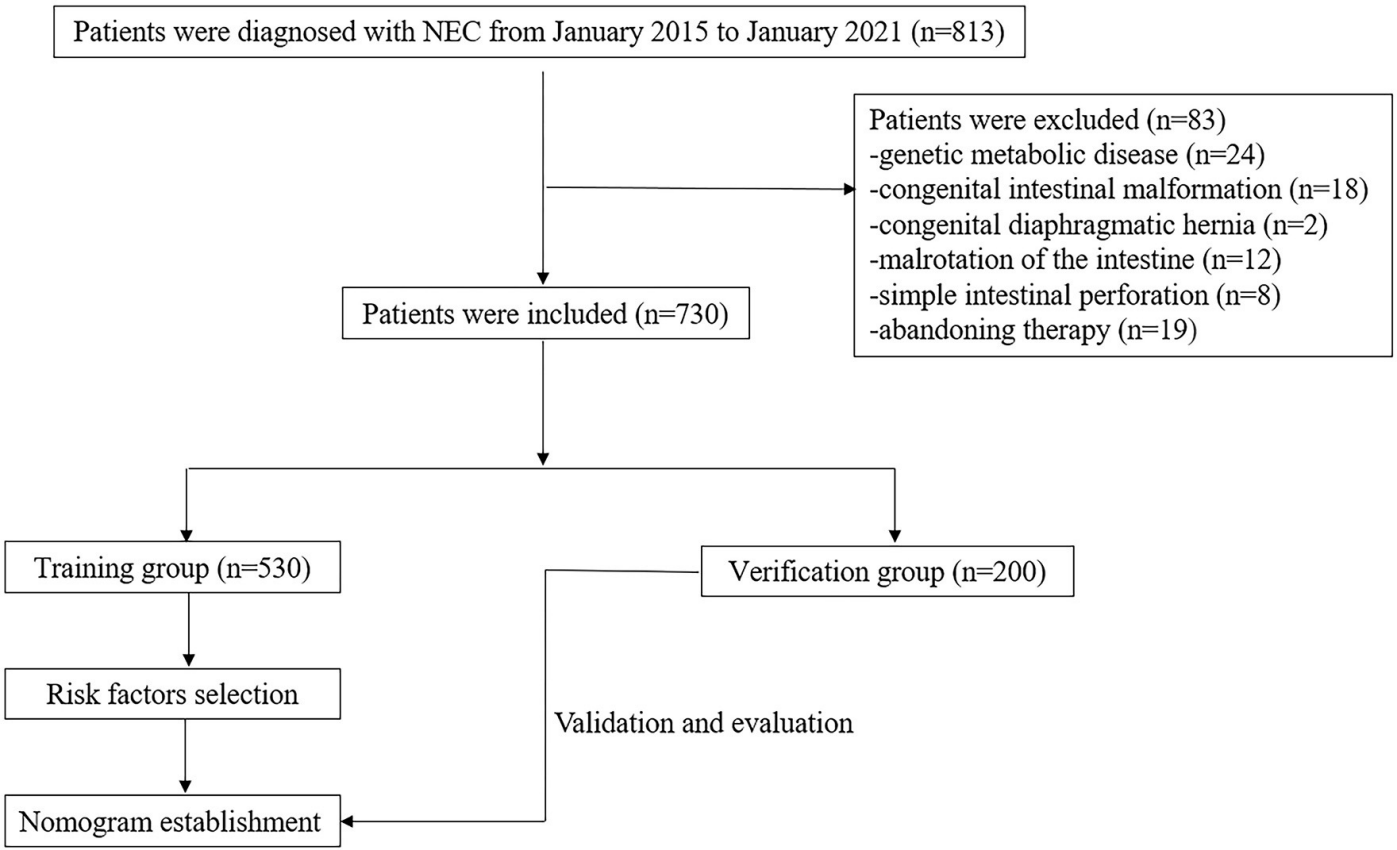
Flowchart of patients included and excluded from the study.

We retrospectively analyzed infants with NEC hospitalized in Anhui Provincial Children's Hospital from January 2015 to January 2021. In total, 730 patients were included; a random sample of 530 newborns was evaluated to construct the nomogram, and the remaining 200 neonates were evaluated for the validation of the model. The flowchart of this study is illustrated in Figure 1. The diagnosis of NEC was based on the Bell and Walsh grading criteria, which includes Stage I, Stage II, and Stage III NEC (13). Infants with stages I and II were defined as suspected and defined NEC, respectively. And those with stage III were defined as having advanced NEC. In this study, stages ≥II NEC were considered a positive outcome. Patients with serious systemic diseases were excluded, such as immune deficiency disease, genetic metabolic disease, pulmonary hemorrhage, congenital intestinal malformation, congenital diaphragmatic hernia, malrotation of the intestine, meconium intestinal obstruction, and simple intestinal perforation. Patients with incomplete medical records were also excluded. This study was approved by the ethics review board of Anhui Provincial Children's Hospital (Nr: EYLL-2017-023).

### Variables and Definitions

The clinical data obtained were from the patient's medical history during hospitalization. The variables collected were as follows: gender, GA, BW, conception method, gestational births, small for GA (SGA), delivery method, anemia in pregnancy, gestational hypertension, hypothyroidism in pregnancy, gestational diabetes mellitus (GDM), erythrocyte infusion, premature rupture of the membrane (PROM), asphyxia, feeding method, delayed fecal excretions, neonatal anemia, coagulation disorders, cerebral hemorrhage, hypoxic-ischemic encephalopathy (HIE), septicemia, hypoglycemia, hypoalbuminemia, PDA, patent foramen ovale (PFO), neonatal scleroderma, and hyperbilirubinemia.

The definitions of gestational hypertension, GDM, and hypothyroidism in pregnancy were in accordance with the criteria described in Williams Obstetrics (14). Delivery method was defined as the way in which the fetus leaves the mother's uterus, including vaginal and cesarean sections. The feeding method was defined as the type of feeding the infant after birth. SGA was defined as a BW lower than the 10th percentile for the GA (15). Hypoglycemia was defined as a blood glucose level <2.6 mmol/L after birth (16). PROM was defined as a spontaneous rupture of embryonic membrane occurring before the onset of labor. Asphyxia was defined as the failure to initiate or sustain spontaneous breathing at birth, a 1-min Apgar score ≤ 7 and cord umbilical arterial pH <7.15. Delayed fecal excretion was defined as the fetus not passing stool for more than 24 h. Anemia was defined as peripheral blood Hb ≤ 145 g/L within 2 weeks of birth, and the peripheral blood Hb ≤ 110 g/L within 2 weeks to 1 month of birth (17). Coagulation disorder was defined as a prothrombin time (PT) prolonged by more than 3 s and an activated partial prothrombin time (APTT) prolonged by more than 10 s. Cerebral hemorrhage was defined as signs of bleeding that were present on imaging examinations. HIE was defined as umbilical cord arterial blood obtained at delivery (pH <7.00 and base deficit of 12 mmol/L or higher), Apgar scores of 0–3 beyond 5 min and early imaging showing evidence of acute nonfocal cerebral abnormality (18). Septicemia was defined as laboratory tests showing WBC > 25 × 10^9^/L for <3 d or WBC > 20 × 10^9^/L for > 3 d, the number of immature/total neutrophils was ≥0.16, or the C-reactive protein was ≥8 mg/L and the presence of clinical manifestations, such as an abnormal body temperature, reduced feeding, less crying, and poor responses. The gold standard for septicemia is a positive blood or cerebral spinal fluid culture. Hypoalbuminemia was defined as blood albumin levels <30 g/L. PDA was defined ASA diameter of the ductus arteriosus that was ≥1.5 and left atrium to aortic valve (LA: AO) ratio of ≥1.5 (19). Neonatal scleroderma was defined as the presence of hypothermia (≤ 35°C) and swollen skin. Hyperbilirubinemia was defined as serum bilirubin levels exceeding the 95th percentile according to the Bhutani curve (20).

### Statistical Analysis

All statistical analyses were processed by SPSS (version26.0) and STATA (version 16.0). Categorical variables were presented as whole numbers and percentages (%), and the chi-square test was used. Variables with a *p* level <0.05 in the univariate analysis were entered into the logistic regression model for the multivariate analysis. A final model was constructed using a backward step-down process, which was based on Akaike's information criterion as the stopping rule. Finally, these potential predictors were used to build a nomogram model. A *p-*value of <0.05 was considered to be statistically significant.

## Results

### Clinical Characteristics of the Subjects

A total of 730 patients were enrolled in this study. The characteristics of the included patients are summarized in Table 1. The training group consisted of 530 (72.6%) neonates, including 229 neonates with Stage I NEC and 301 neonates with Stage II /III NEC. The validation group consisted of 200 (27.4%) neonates, with 75 Stage I NEC neonates and 125 Stage II or III NEC neonates. The infants in the two groups had some similar clinical characteristics. There was no statistical difference in gender, conception method, gestational births, delivery method, anemia in pregnancy, hypothyroidism in pregnancy, GDM, PROM, feeding method, delayed fecal excretion, HIE, PFO, or neonatal scleroderma between the two groups (*p* > 0.05). While GA, BW, hypertension in pregnancy, erythrocyte infusion, asphyxia, neonatal anemia, coagulation disorders, cerebral hemorrhage, septicemia, hypoglycemia, hypoalbuminemia, PDA, and hyperbilirubinemia were statistically significant between the two groups(*p* <0.05) (Table 1).

**Table 1:** Characteristics of the included patients.

	**Training group (*****n*** **=** **530)**		**Verification group (*****n*** **=** **200)**		
	**Stage I NEC** **(*n* = 301)**	**Stage II/III NEC** **(*n* = 229)**	**Stage I NEC** **(*n* = 125)**	**Stage II/III NEC** **(*n* = 75)**	***p-value***
Gender (%)					0.940
Male	187(62.13%)	143(62.45%)	71(56.80%)	44(58.67%)	
Female	114(37.87%)	86(37.55%)	54(43.20%)	31(41.33%)	
BW (g)					<0.001
<1500	17(5.65%)	91(39.74%)	5(4.00%)	32(42.67%)	
1500–2500	64(21.26%)	63(27.51%)	24(19.20%)	20(26.67%)	
>2500	220(73.09%)	75(32.75%)	96(76.80%)	23(30.67%)	
GA (weeks)					<0.001
<32	25(8.31%)	102(44.54%)	75.60%	27(36.00%)	
32–37	68(21.26%)	57(24.89%)	29(23.20%)	28(37.33%)	
>37	208(69.10%)	70(30.57%)	89(71.20%)	20(26.67%)	
SGA(%)	48(15.95%)	40(17.47%)	19(15.20%)	18(24.00%)	0.641
Conception method (%)					0.287
Natural conception	293(97.37%)	219(95.63%)	121(96.80%)	71(94.67%)	
*in vitro* fertilization	8(2.66%)	10(4.37%)	4(3.20%)	4(5.33%)	
Gravida (%)					0.946
Single	257(85.38%)	196(85.59%)	110(88.00%)	60(80.00%)	
Twins	44(14.62%)	33(14.41%)	15(12.00%)	15(20.00%)	
Delivery method (%)					0.753
Vaginal	147(48.84%)	115(50.22%)	69(55.20%)	35(46.67%)	
Cesarean section	154(51.16%)	114(49.78%)	56(44.80%)	40(53.33%)	
Anemia in pregnancy (%)	14(4.65%)	13(5.68%)	11(8.80%)	2(2.67%)	0.595
Hypertension in pregnancy (%)	19(6.31%)	28(12.23%)	7(5.60%)	11(14.67%)	0.020
Hypothyroidism in pregnancy (%)	10(3.32%)	7(3.06%)	7(5.60%)	0(0.00%)	0.864
GDM (%)	17(5.65%)	12(5.24%)	10(8.00%)	3(4.00%)	0.838
Erythrocyte infusion (%)	37(12.29%)	81(35.37%)	14(11.20%)	31(41.33%)	<0.001
PROM (%)	56(18.6%)	57(24.89%)	27(21.60%)	13(17.33%)	0.081
Asphyxia (%)	26(8.64%)	41(17.90%)	7(5.60%)	9(12.00%)	0.002
Feeding method (%)					0.661
formula feeding	189(62.79%)	159(69.43%)	72(57.60%)	53(70.67%)	
breastfeeding	68(22.59%)	28(12.23%)	41(32.80%)	8(10.67%)	
no feeding initiation	44(14.62%)	42(18.34%)	12(9.60%)	14(18.67%)	
Delayed fecal excretion (%)	14(4.65%)	19(8.30%)	13(10.40%)	4(5.33%)	0.089
Neonatal anemia (%)	55(18.27%)	97(42.36%)	20(16.00%)	29(38.67%)	<0.001
Coagulation disorders (%)	23(7.64%)	36(15.72%)	7(5.60%)	10(13.33%)	0.004
Cerebral hemorrhage (%)	57(18.94%)	64(27.95%)	18(14.40%)	19(25.33%)	0.015
HIE (%)	7(2.33%)	4(1.75%)	3(2.40%)	1(1.33%)	0.644
Septicemia (%)	30(9.97%)	90(39.30%)	12(9.60%)	23(30.67%)	<0.001
Hypoglycemia (%)	28(9.30%)	90(39.30%)	8(6.40%)	21(28.00%)	<0.001
Hypoalbuminemia (%)	10(3.32%)	24(10.48%)	1(0.80%)	10(13.33%)	<0.001
PDA (%)	20(6.64%)	65(28.38%)	4(3.20%)	26(34.67%)	0.002
PFO (%)	34(11.30%)	33(14.41%)	17(13.60%)	11(14.67%)	<0.001
Neonatalscleroderma (%)	4(1.33%)	9(3.93%)	0(0.00%)	1(1.33%)	0.286
Hyperbilirubinemia (%)	157(52.16%)	83(36.24%)	59(47.20%)	36(48.00%)	0.067

*Values in parentheses are percentages; BW, birth weight; G, gestational age; SGA, small for gestational age; GDM, gestational diabetes mellitus; PDA, patent ductus arteriosus; PFO, patent foramen ovale; HI, hypoxic-ischemic encephalopathy; PROM, premature rupture of membrane*.

### Multivariate Regression Analysis

Multivariate logistic regression analysis identified six variables as independent risk predictors in the training group: BW <1,500 g (OR:4.057, 95%CI = 1.390–11.837), GA <32 w (OR:3.457, 95%CI = 1.275–9.469), asphyxia (OR:2.037, 95%CI = 1.051–3.945), septicemia (OR:5.563, 95%CI = 3.194–9.690), hypoglycemia (OR:4.279, 95%CI = 2.464–7.431), PDA (OR:2.863, 95%CI = 1. 492 −5.388). The details are summarized in Table 2.

**Table 2:** Multivariate regression analysis of significant risk factors.

**Variables**	***B***	**S.E**.	**Wald**	**OR (95%*CI*)**	***p-*value**
GA (weeks)			6.159		0.046
<32	1.254	0.512	5.929	3.457 (1.275–9.469)	0.015
32–37	0.561	0.328	2.920	1.753 (0.921–3.336)	0.087
BW (g)			6.793		0.033
<1500	1.400	0.546	6.569	4.057 (1.390–11.837)	0.010
1500–2500	0.357	0.339	1.111	1.429 (0.736–2.777)	0.292
Neonatal asphyxia (%)	0.711	0.337	4.444	2.037(1.051–3.945)	0.035
Septicemia (%)	1.716	0.283	36.734	5.563 (3.194–9.690)	<0.001
Hypoglycemia (%)	1.454	0.282	26.639	4.279 (2.464–7.431)	<0.001
PDA (%)	1.042	0.328	10.127	2.836 (1.492–5.388)	<0.001

*BW, birth weight; GA, gestational age; PDA:, patent ductus arteriosus*.

### A Nomogram Prediction Model Construction

**Figure 2:**
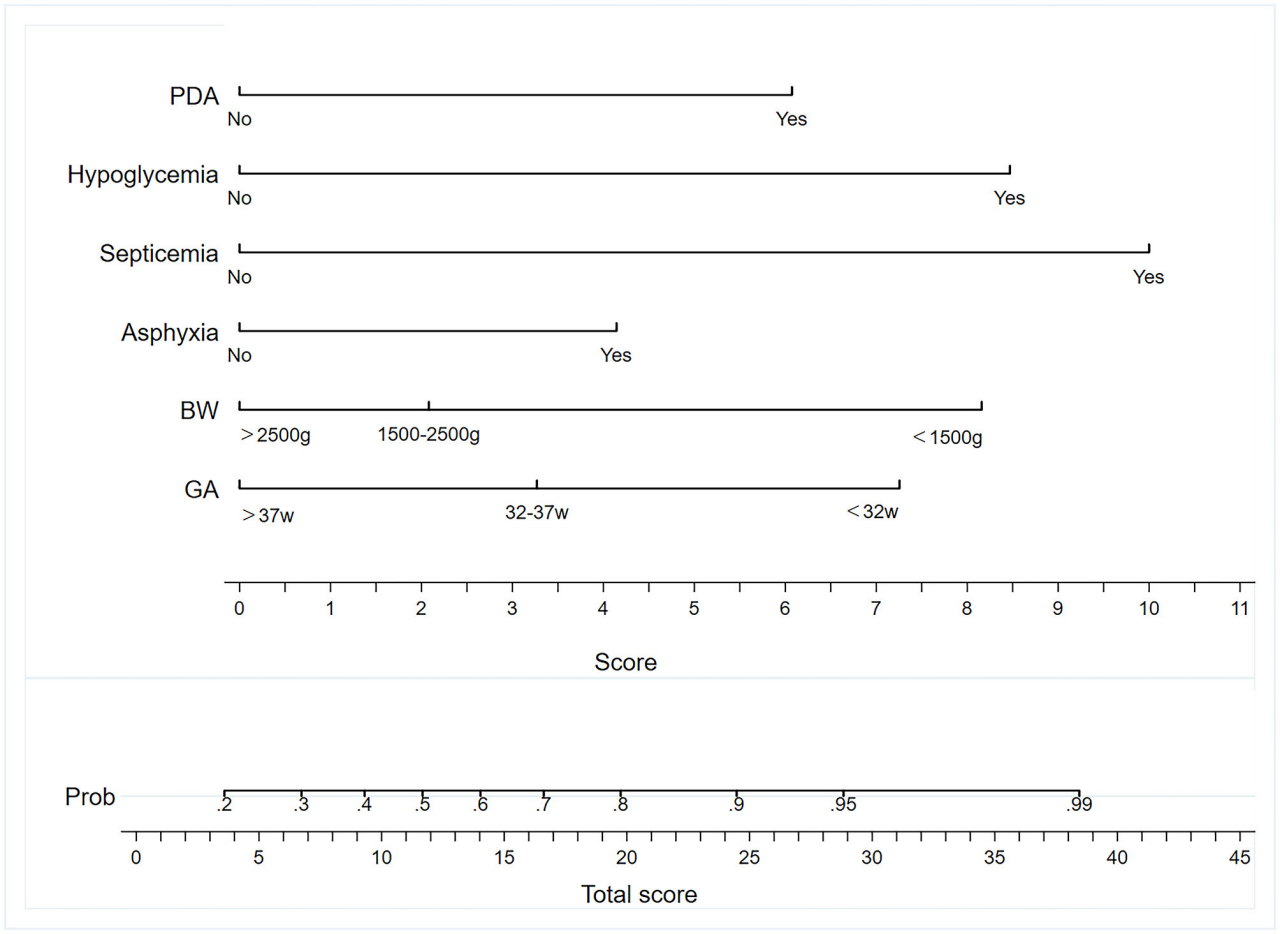
Nomogram to predict the incidence of NEC. BW, birth weight; GA, gestational age; PDA, patent ductus arteriosus.

A nomogram model containing the six variables mentioned above was established (Figure 2). Each variable had different corresponding scores. A score of 6.2 is in the presence of a PDA, 10.4 for sepsis, 8.5 for hypoglycemia, 4.2 for asphyxia, 7.5 for GA <32 w, and 8.3 for BW <1,500 g. For patients with suspected NEC whose GA is <32 w and BW was <1,500 g, and who had PDA and asphyxia, we achieved corresponding scores approximately of 7.5, 8.3, 6.2, 4.2, 0, and 0 after drawing a vertical line on the corresponding point. The score of all the variables (when added together) was 26.2. Then, the probability for stage II/III NEC that we obtained was approximately 91% when we drew a vertical line on the corresponding point.

### Validation and Evaluation of the Nomogram Model

**Figure 3:**
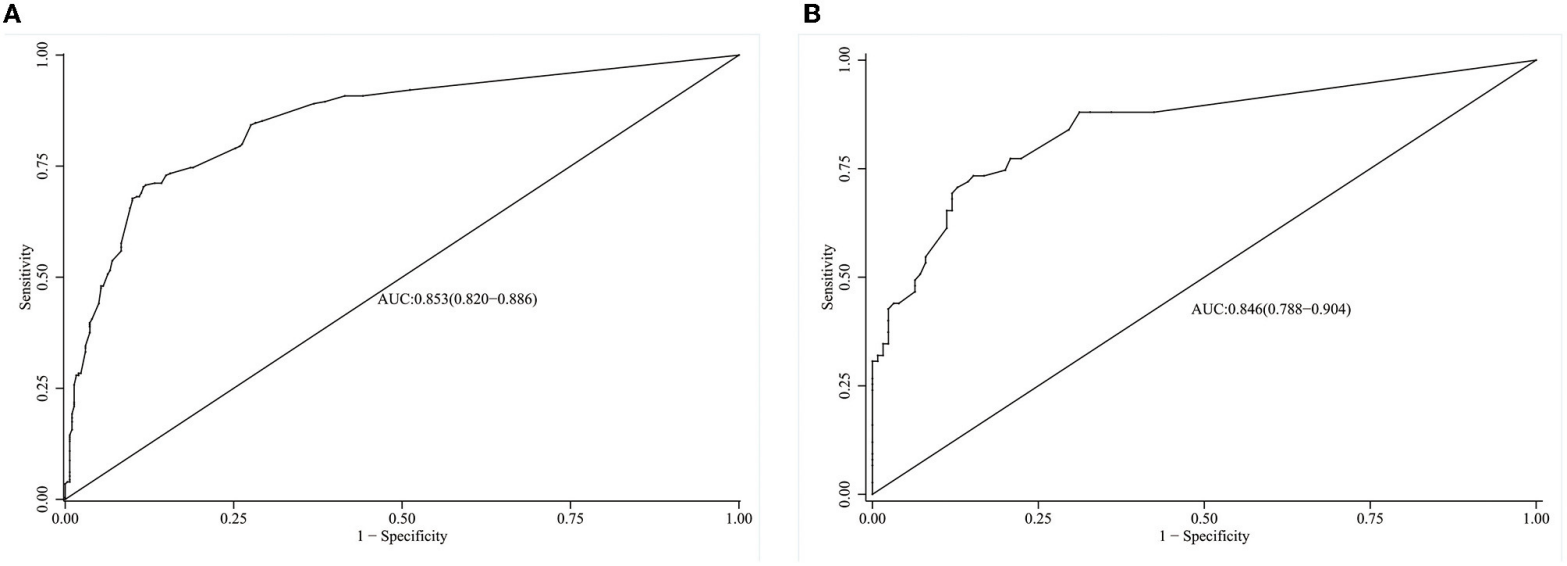
ROC curves of the model in the training and validation group. **(A)** ROC of the model in the training group. **(B)** ROC of the model in the validation group.

The model's degree of discrimination was evaluated using the receiver operating characteristic curve (ROC), with a higher AUC representing a better degree of discrimination. The AUCs were 0.853 (95% CI, 0.82–0.89) and 0.846 (95% CI, 0.79–0.90) in the training and verification groups, respectively, which indicated that the model had a good discrimination ability (Figures 3A,B).

**Figure 4:**
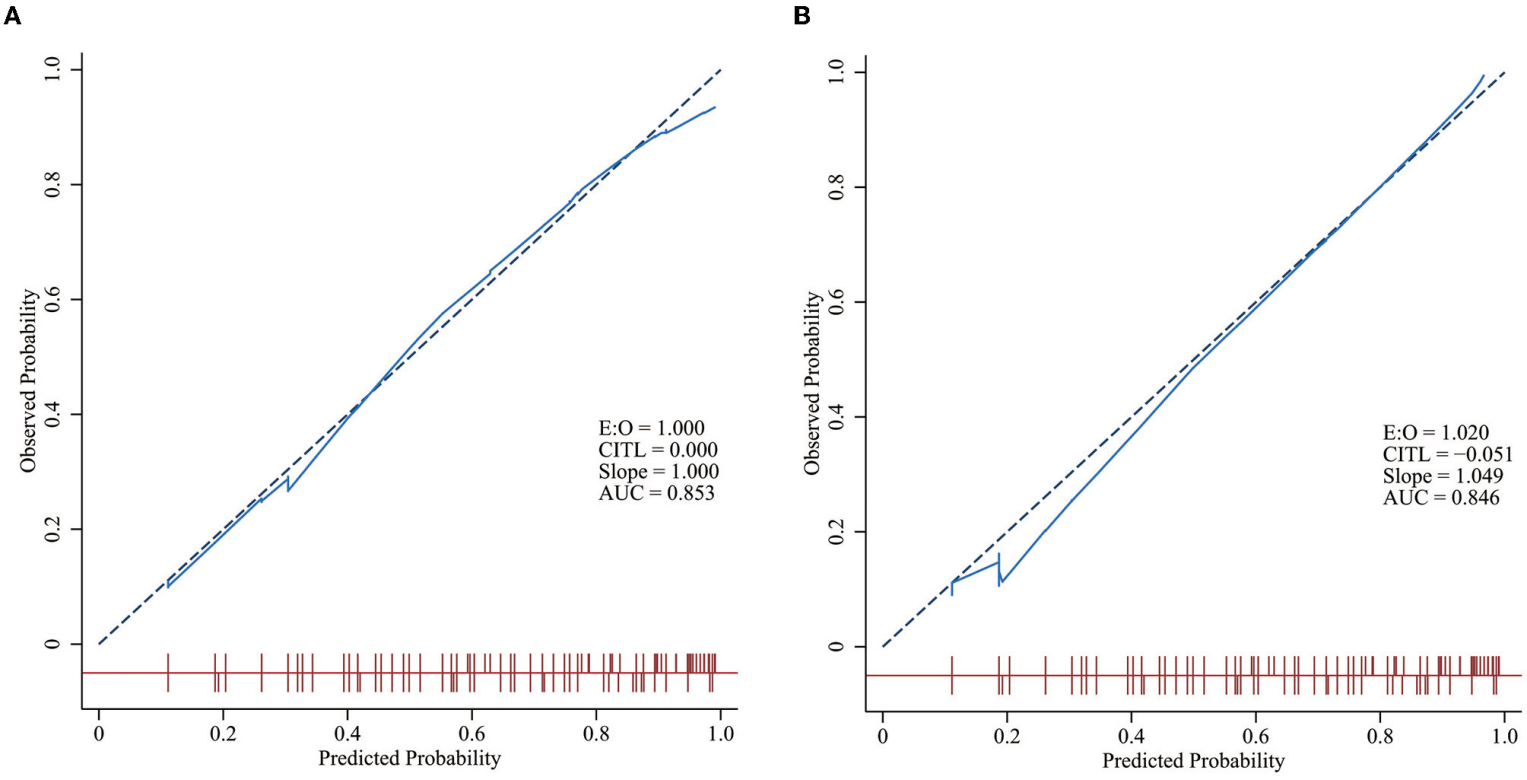
Calibration curves of the models in the training and validation groups. The closer the solid blue line is to the red dashed line with a slope of one, the better the model is calibrated. A level *p* > 0.05 in the two figures indicates no statistically significant difference between the predicted incidence curve and the actual incidence curve. **(A)** Calibration curves of the model in the training group. **(B)** Calibration curves of the model in the validation group.

The calibration curve is an important index for assessing the calibration of the model, and it reflects the degree of consistency of a nomogram. There was no statistically significant difference between the risk prediction value and the actual risk for NEC based on the nomogram for NEC (χ^2^ =8.238, *p* = 0.221), indicating that the model had good prediction consistency (Figures 4A,B).

**Figure 5:**
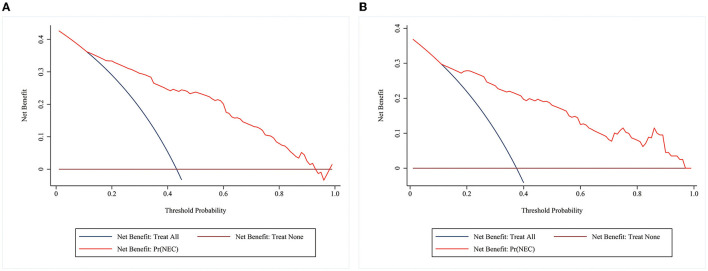
DCA curves of the model in the training and validation groups. The horizontal coordinate is the threshold probability. The vertical coordinate is the net benefit after subtracting the harm (misdiagnosis) from the benefit (the benefit is for the patient to be treated). The brown reference line indicates that all patients don't receive any intervention, and the net benefit is zero. The dark blue reference line indicates that all patients receive treatment, and the net benefit is represented by the backslash of the slope. The further the model curve is away from the two reference lines, the better the clinical utility of the nomogram. **(A)** DCA of the model in the training group **(B)** DCA of the model in the validation group.

A decision curve analysis (DCA) curve was utilized to evaluate the clinical utility of the prediction model. Our study showed that the nomogram had a high level of clinical net benefit when the prediction probability threshold was between 0.12 and 0.93 in the training group, suggesting that the nomogram model had good clinical utility (Figures 5A,B).

## Discussion

In this retrospective cohort study, we calculated and validated a personalized prediction nomogram model that predicted the risk of Bell's stage II/III NEC, which is useful for clinicians when making treatment plans for infants with NEC. Finally, we identified six associated risk factors: GA, BW, PDA, septicemia, asphyxia, and hypoglycemia. There is no doubt that GA and BW should be included in the model, and both have been shown to be strongly associated with NEC in many studies [1, 2, 5, 6, and (21)]. Our research results also confirmed this idea. The possible factors involved in the development of NEC in infants with a lower GA are the immaturity of the gastrointestinal tract, immune defense, circulation regulation, digestive function, and barrier function (22).

Septicemia is recognized as a risk predictor for NEC. When sepsis occurs, a large number of bacteria can multiply in the blood, produce toxins, and cause damage in the intestine. However, the newborn's intestine is immature. Hence, exposure to the above factors can lead to the release of a large number of cytokines, causing inflammation in the body, which promotes necrosis of the intestinal mucosa, and damages the intestinal barrier function (23). It has been reported that the incidence of NEC in newborns with septicemia ranges from 34 to 57% (24–26). All patients suspected of sepsis should receive antibiotics basing guidelines, but long-term exposure to antibiotics may increase the occurrence of NEC (27), especially third-generation cephalosporins (28). Western countries prefer to use ampicillin and aminoglycoside antibiotics, so the use of antibiotics in neonates with sepsis requires reasonable choice.

In mice models, NEC can be induced by intermittent hypoxia and giving formula milk feeding (29). Previous clinical studies have shown that hypoxia can lead to NEC in infants (26). Our data support the results of studies that have described an association between NEC and asphyxia. This association may be due to the imbalance between oxidation and anti-oxidation in the body under low oxygen. Aydemir et al. (30) found that the levels of total antioxidant capacity and oxidative stress index correlated with the severity of NEC were significantly elevated. Therefore, newborns with asphyxia after birth need to need to be monitored for abdominal signs and treated in a timely manner to reduce the gastrointestinal burden, if necessary.

In our study, PDA was correlated with NEC, which was similar to the results of previous studies (7). This could be due to a reduced intestinal blood flow during catheter shunting. Prophylactic surgical ligation of the PDA was associated with significant reduction in the incidence of stage II or III NEC (31), but it is an invasive operation. Currently, people are paying increasing attention to medical therapy for PDA cases. A Cochrane systematic review concluded that ibuprofen and indomethacin were helpful for PDA closure, and could reduce the risk of NEC and transient renal insufficiency (32). However, there is currently no consensus on whether PDA in preterm infants requires treatment, how, and when these infants should be treated.

Hypoglycemia was also found to be a predictive factor in this study, and hypoglycemia is common in neonates. It may be caused by a variety of diseases, such as sepsis, hypermetabolic diseases, hyperinsulinemia, fatty acid oxidative disorders, gluconeogenic disorders, and glycogen deficiency (33). Studies have shown that hypoglycemia secondary to hypopituitarism increased the risk for NEC (34). In addition, 46 of the 118 patients with hypoglycemia in the training set also had sepsis in this study. However, there are few studies on the relationship between hypoglycemia and NEC, and further research is needed

In recent years, some researchers have linked erythrocyte infusions with the development of NEC in infants and have hypothesized that erythrocyte infusion could be a cause of NEC (35). The possible mechanisms of NEC induced by blood transfusion are as follows: (1) Blood transfusions cause hemodynamic instability and uneven distribution of intestinal blood supply, leading to ischemia, and necrosis of immature intestinal mucosa. (2) It may be similar to transfusion-related acute lung injury (TRALL), the host by primary disease induces vascular endothelial activation, chemokine release, and neutrophil activation, when biological immune response factors, such as Human Leukocyte Antigen (HLA) antibodies, bioactive substances, free hemoglobin, red blood cell debris, and inflammatory cytokines present in the blood promote a cascade reaction between neutrophils and the complement system causing secondary damage (36). (3) Reduced NO in stored blood leads to vasoconstriction and reduced intestinal blood flow and oxygenation (37). However, some studies have shown that there is no association between red blood cell transfusions and NEC (38), and some reports have even suggested that these transfusions could decrease the risk of NEC (39). Therefore, the relationship between erythrocyte infusions and the occurrence of NEC is not very clear. In our study, transfusion did not increase the risk of NEC. Moreover, formula feeding in this study was not included in the model as a risk factor for NEC, which could be related to the low breastfeeding rate in our hospital and the limitations of the sample size utilized. In the past 2 years, a milk donation bank was established in our hospital, and feeding method including breast milk, donor milk, and formula will be further evaluated in prospective studies.

A nomogram is a graph drawn by turning the regression coefficients into scores after appropriate mathematical operations, and its advantages are strongly visualized. And the degree of correlation between each variable and outcome could be directly reflected by the score. Clinicians could early identify stage II/III NEC early through this nomogram and calculate the probability of occurrence. If the probability is very high, the doctor needs to take appropriate treatment measures in time. In severe cases, it is even necessary to recommend surgery for infants, which helps avoid missing the optimal treatment time. But the nomogram looks complicated at first glance, and you may need to learn how to use it.

Several limitations exist in this study. Our clinical data originated from a single hospital, and the presence of selection bias cannot be ignored. Moreover, it may also be difficult to determine whether the study applies to patients in other countries, considering the racial differences (40), and further external verifications are needed. In future research, we plan to develop other clinical prediction models for NEC, such as models based on CART and ANNs. By comparing the advantages and disadvantages, we will select the best model for NEC prediction. Then, we will carry out further multicenter, prospective studies to validate the optimal model for generalization.

## Conclusion

We have developed and internally validated a novel nomogram model for predicting the risk of Bell's stage II/III NEC compared to Bell's stage I NEC. The nomogram with the advantages of being easy to use and having an excellent calibration might help clinicians to make early individualized predictions of each patient's probability of Bell's stage II/IIINEC early and to take early therapeutic measures, thus reducing the severity of NEC. To our knowledge, we have established the first risk assessment tool for NEC.

## Data Availability Statement

The original contributions presented in the study are included in the article/supplementary material, further inquiries can be directed to the corresponding author/s.

## Ethics Statement

The studies involving human participants were reviewed and approved by Anhui Provincial Children's Hospital. Written informed consent to participate in this study was provided by the participants' legal guardian/next of kin.

## Author Contributions

LD and SS conceived and designed the study. SS collected and sorted out the data and was responsible for drafting the manuscript. JZ guided the data statistical analysis. JZ and YZ revised the manuscript. All authors contributed to the article and approved the submitted version.

## Conflict of Interest

The authors declare that the research was conducted in the absence of any commercial or financial relationships that could be construed as a potential conflict of interest.

## Publisher's Note

All claims expressed in this article are solely those of the authors and do not necessarily represent those of their affiliated organizations, or those of the publisher, the editors and the reviewers. Any product that may be evaluated in this article, or claim that may be made by its manufacturer, is not guaranteed or endorsed by the publisher.
